# Preoperative animated videos reduce education time and increase content awareness for patients with digital subtraction angiography-guided implantable venous access ports

**DOI:** 10.1097/MD.0000000000040486

**Published:** 2024-11-15

**Authors:** Zhexia Jin, Zhongfeng Niu, Chunqiao Wu, Xi Hu, Funv Shen, Yayun Xiao, Yan Zhang

**Affiliations:** a Nursing Department, Sir Run Run Shaw Hospital, Zhejiang University School of Medicine, Hangzhou, China; b Department of Radiology, Sir Run Run Shaw Hospital, Zhejiang University School of Medicine, Hangzhou, China.

**Keywords:** animation, health education, intravenous infusion, nursing clinical research

## Abstract

This study aimed to discuss the clinical value of health education using an animated video for postoperative patients with digital subtraction angiography (DSA)-guided implantable venous access ports. Retrospective study. Based on expert consensus and clinical experience, we created an animated video presenting postoperative health education for patients to watch after infusion port implantation and uploaded it to a multimedia room. A total of 93 patients who underwent DSA-guided implantable venous access port placement at our hospital from March to June 2022 and from July to October 2022 were selected. Forty-six patients who received traditional oral and written education from March to June 2022 were selected as the control group. Forty-seven patients who received animated video-assisted health education from July to October 2022 were selected as the experimental group. The time spent on health education and patients’ awareness of the educational content were compared between the 2 groups. The time spent on health education in the experimental group was 3.51 ± 0.62 minutes, which was less than that of the control group, 6.76 ± 1.14 minutes (*t* = 17.07, *P* < .001). Patients’ awareness of educational content in the experimental group was 8.62 ± 1.26 points, which was significantly better than the control group’s 7.26 ± 1.63 points (*t* = −4.490, *P* < .001). Animated video-assisted health education can enable patients to gain a better understanding of educational content in a shorter time. Moreover, it can improve nurses’ interventional efficiency. Therefore, this health education method is worthy of clinical promotion.

## 1. Introduction

An implantable venous access port (IVAP) is a closed infusion device that is completely implanted in the body and can be used for long-term infusion of highly concentrated and irritating drugs.^[1–3]^ It is widely used because, compared to other central venous access devices (i.e., non-tunneled central venous catheters [CVCs], peripherally inserted CVCs, and tunneled CVCs), it has aesthetic advantages, as well as the advantages of a low drug extravasation rate, low infection rate, and a long maintenance interval.^[4,5]^

Postoperative health education after infusion port implantation aims to inform patients about the postoperative position and puncture site, infusion port maintenance, post-discharge guidance, and related precautions.^[6,7]^ Studies have shown that effective health education can directly affect postoperative recovery.^[8,9]^

Various forms of postoperative health education, such as interviews, have been reported, most of which consist of oral or written explanations.^[10–12]^ However, there are disadvantages to this, such as difficulty in understanding, a high rate of forgetting, and boring text content.^[13]^ Moreover, most patients have limited medical backgrounds and have difficulty absorbing relevant postoperative knowledge within a short period.

Studies have demonstrated video applications of preoperative education on infusion port implantation, including therapeutic regimens, types of infusion ports, surgical procedures, and intraoperative cooperation. However, there are only a few video applications regarding 24-hour post-infusion port implantation, during treatment, and home care guidance.^[14]^ Meanwhile, with the change in China’s medical policies, infusion port implantation-related medical materials have been included in the basic medical insurance catalog, which has led to a dramatic increase in their clinical use. Consequently, postoperative health education work requires nurses to invest a lot of their time and energy. Therefore, it is crucial to develop a rational and efficient postoperative health education model. Our group produced an animated video, a relatively novel and attractive media, about the precautions to be taken after infusion port implantation. We then applied this to postoperative health education.

This study aimed to investigate the superiority of animated video-assisted postoperative health education by comparing its effectiveness with traditional oral and written postoperative health education explanations for patients after infusion port implantation.

## 2. Methods

### 2.1. Study design

This retrospective study was designed to evaluate the effectiveness of animated videos in health education for postoperative patients using a digital subtraction angiography (DSA)-guided IVAP.

### 2.2. Participants

We reviewed the records of patients who underwent infusion port implantation at our hospital. The inclusion criteria were indications for infusion port implantation, clear consciousness, barrier-free language communication, and basic listening, speaking, reading, and writing skills. Patients with a medical history or mental illness were excluded from the study.

### 2.3. Sample size

We considered health education to be face-to-face oral or written explanation, before March 2022. During this period, we did not record the patients’ awareness of the educational content or the time spent on health education; subsequently, we noted this issue and began collecting data in March 2022. Through this process, we realized that the patients had a poor understanding of the health education content after infusion port implantation. Therefore, we produced an animated video of the precautions to be taken after infusion port implantation, applied it to postoperative health education beginning in July 2022, and collected the relevant data. Ultimately, 93 patients were included in this study. A sum of 46 patients between March and June 2022 and 47 patients between July and October 2022 were included in the control and experimental groups, respectively.

### 2.4. Venous access port implantation procedure

The implantation procedure was as follows: The patient was placed in a supine position. Preoperative marking and disinfection were performed, and local infiltration anesthesia was administered. A posterior approach puncture in the internal jugular vein was administered, guided by ultrasound, and the guidewire was placed. The peel-away sheath was exchanged, and the catheter was inserted. A pocket of about 1 at 2 cm below the clavicle of the chest wall was established. A tunneling device was used to guide the catheter from the neck incision to the pocket. The catheter and the infusion port were connected, and the catheter length was adjusted according to the X-ray. The incision was closed. Finally, the needle puncture was made, and the catheter was locked with heparin saline. All postoperative health education work was completed by the same nurse (with > 5 years of interventional work experience). The hospital ethics committee reviewed the study protocol.

### 2.5. Intervention methods for the experimental group

#### 2.5.1. Study group establishment

The study group had 6 members. The departmental head nurse served as the team leader and was responsible for formulating the duties and the overall planning and implementation of the project. Nurse A was responsible for patient education. This nurse was selected based on the following criteria: an intermediate title or above, good communication skills, proficiency in catheterization room workflow, rules, and regulations, and a comprehensive understanding of postoperative health education content, especially regarding post-infusion port implantation. Nurse B was responsible for the data collection and analysis. Nurse C produced the animated videos. Nurse C’s selection was based on the following criteria: a keen interest in collecting pictures, animations, and audio materials and an ability to make full use of computer technology to design artistic images to present medical knowledge in the form of animation. An information technology engineer was responsible for playing the animated video in the department’s multimedia room. The director of the interventional department (a senior physician) served as a project consultant and was responsible for quality supervision throughout the process.

#### 2.5.2. Animated video content determination

The animated video content was developed based on expert consensus, product specifications, clinical experience,^[6,15]^ and other relevant information. This included the following: First, during the 24-hour post-operation period, patients were instructed to try to rest in bed and reduce activities, adopt a supine or healthy-side lateral position, maintain a healthy postoperative diet, and monitor soreness at the puncture site, which should self-resolve in 1 to 2 days. They were also told that the infusion could be administered immediately after surgery. Second, treatment instructions included directions to wear loose clothing, avoid strenuous exercise, replace the butterfly needle pathway management during the infusion, and take precautions for CT and MRI examinations. Third, instructions for home care guidance included the need to observe the wound and seek prompt medical treatment in the presence of any abnormalities. Other instructions were related to exercise guidance, daily maintenance, and removal of the infusion ports.

#### 2.5.3. Animated video creation

**Figure 1.**
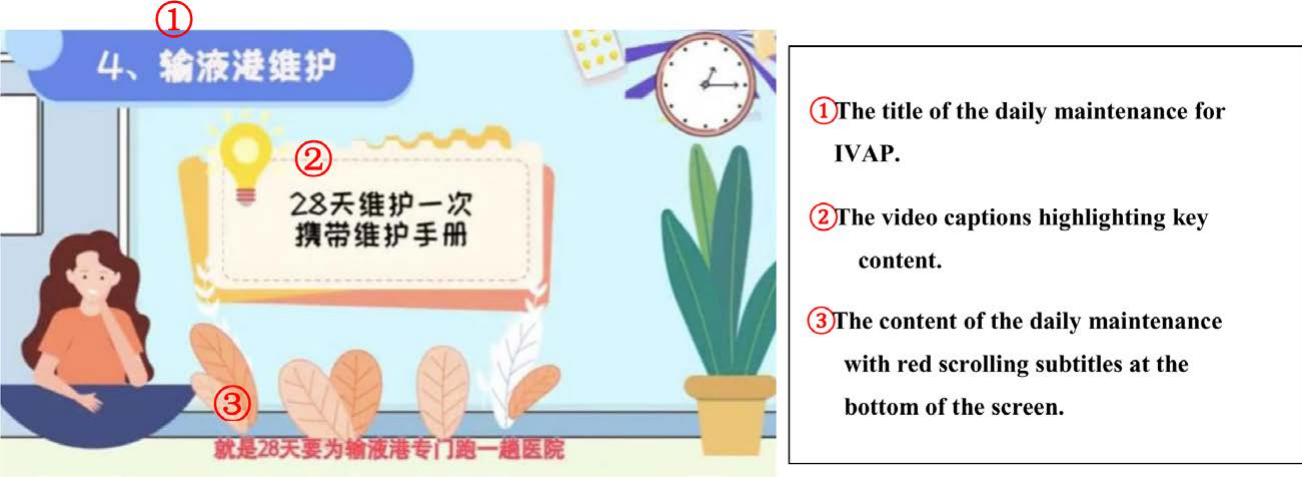
The screen of animated video “daily maintenance for implantable venous access port”. IVAP = implantable venous access port.

The video was produced online on a computer. First, the theme of “postoperative health education” was introduced by an interventional nurse who noticed that patients displayed anxiety and confusion after infusion port implantation. Planning was designed according to the video content, and the video script was written in easy-to-understand language. Interventional nurses were selected during the creation of these videos. During video editing, animated materials were added, followed by post-production synthesis with red scrolling lyrics presented at the bottom of the screen and video captions highlighting key content. Finally, a gentle voice representative of popular science was used for the video narration, and slow background music was added. After the initial draft was completed, the head nurse organized a team meeting to promptly address existing problems before the video adoption. The completed animated video lasted for 4 minutes and 53 seconds, as shown in Figure 1.

#### 2.5.4. Specific implementation process for animated video-assisted postoperative health education

The multimedia room was located near the catheterization room and was equipped with computers, projectors, and seating. The environment was quiet, clean, appropriately lit, and warm. When patients and their families arrived in the catheterization room, the interventional nurse guided them to the multimedia room to repeatedly watch the animated video. They were also informed that following the WeChat channel by scanning the QR code would allow them to watch the video anytime and anywhere. After surgery, the nurse distributed brochures and verbally educated the patients on a one-on-one basis. During the patient education process, the nurse spoke in a calm tone, patiently answered questions about information the patients did not understand until they had fully grasped the concepts, and concluded the session when no more questions were asked.

### 2.6. Intervention methods for the control group

Patients in the control group received the standard method of education without the animated video. The nurse distributed brochures and verbally educated the patients one-on-one after the surgery. During the patient education process, the nurse spoke in a calm tone, patiently answered questions about information the patients did not understand until they had fully grasped the concepts, and concluded the session when no more questions were asked.

### 2.7. Main research tools

#### 2.7.1. The research participants’ basic characteristics

The researchers designed the questionnaire, which included demographic information (sex, age, and education level) as well as the purpose of the IVAP.

#### 2.7.2. Time spent on health education

A timer was used to calculate the time nurses spent on postoperative health education from start to end.

#### 2.7.3. Patients’ awareness of education content

A relevant questionnaire was developed based on the video content, and the expert coordination coefficient was calculated using the Delphi expert consultation method, which indicated good agreement with the experts’ opinions on the selected indicators. The questionnaire had a total of ten questions, and question type (6 single- and 4 multiple-choice) was indicated before each question. The 3 topics were 24-hour post-operation, treatment period, and discharge instructions.

### 2.8. Statistical analysis

SPSS21.0 was selected for data sorting and statistical analysis. The measurement data that were assessed for normality were expressed as mean ± standard deviation, and the t-test of independent samples was employed for between-group comparisons. Counting data were expressed as frequency and percentage, using the *X*^2^ test of independent samples for between-group comparisons. Differences were considered statistically significant at *P* < .05.

### 2.9. Ethical considerations

Research protocols were performed in accordance with the relevant guidelines and regulations. All instruments and methods were approved by the Ethics Committee of the Hospital (approval no. 2022-0247).

## 3. Results

### 3.1. General patient characteristics

Information about sex, age, education level, and purpose of the IVAP was collected for the 2 groups, and there were no significant differences in the general data (*P* > .05), as shown in Table 1.

**Table 1:** Comparison of general data between the 2 groups.

Variables	Cont. (n = 46)	Exp. (n = 47)	Test statistics	*P* value
	n (%) or M ± SD	n (%) or M ± SD		
Sex			0.522*	.470
Men	27 (58.7)	31 (66.0)		
Women	19 (41.3)	16 (34.0)		
Age (yr)	60.48 ± 11.18	61.85 ± 10.99	−0.597†	.552
Education level			0.739‡	.460
Junior college and above	3 (6.5)	4 (8.5)		
Middle school	23 (50.0)	18 (38.3)		
Primary school and below	20 (43.5)	25 (53.2)		
Purpose				>.999
Chemotherapy	46 (100.0)	47 (100.0)		

Cont. = control group, Exp. = experimental group, M = mean, SD = standard deviation.

* *X*^2^ value.

† *t* value.

‡ *Z* value.

### 3.2. Comparison of the time spent on health education and the patients’ awareness of educational content

The time spent on health education and patients’ awareness of educational content were compared between the 2 groups, and the differences were found to be statistically significant (*P* < .05), as shown in Table 2.

**Table 2:** Comparison of time spent for health education and awareness of educational content between the 2 groups.

Variables	Cont. (n = 46)	Exp. (n = 47)	*t* value	*P* value
	n (%) or M ± SD	n (%) or M ± SD		
Time spent on health education (min)	6.76 ± 1.14	3.51 ± 0.62	17.075	<.001
Awareness of educational content (point)	7.26 ± 1.63	8.62 ± 1.26	−4.490	<.001

Cont. = control group, Exp. = experimental group, M = mean, SD = standard deviation.

## 4. Discussion

### 4.1. Animated video-assisted postoperative health education is an effective measure to improve patients’ awareness of education content after infusion port implantation

In this study, patients’ awareness of educational content in the experimental group registered 8.62 ± 1.26 points, which was significantly better than the control group’s 7.26 ± 1.63 points. This is consistent with the findings of experts such as Aisah, Supad, and Platto,^[16–18]^ who found that multimedia forms such as animated videos can enhance the effectiveness of health education.

Postoperative precautions for infusion port implantation are multifaceted. Previously, such information was primarily accessible to patients through pamphlets or verbal indoctrination by surgical nurses. Currently, DSA-guided IVAPs are classified as low-level care, and the related nursing procedures are not complex. Such procedures are typically performed by only 1 nurse, resulting in limited human resources.^[19,20]^ Therefore, the traditional mode of providing information can easily overwhelm patients, making them feel that the information sessions are too short. This directly affects their interest in receiving content, ultimately leading to a decline in compliance and difficulty in comprehending patient education materials.^[21]^

A vivid and easy-to-understand animated video was produced and uploaded to a multimedia room accessible to patients. The video content was developed based on expert consensus and information brochures developed by the department. The content was systematic, comprehensive, standardized, and scientifically sound. In practice, patients and their families can watch the video online according to their needs, either when they arrive at the catheterization room to wait for the procedure or after surgery. Animated videos help patients deepen their understanding through both visual and auditory stimulation.^[22–24]^ Furthermore, by using situational drama as a mode of introduction, videos promote patient empathy and enhance their interest in the content. Additionally, patients do not experience time restrictions when watching videos; therefore, they have more time to learn. This compensates for the shortcomings of traditional methods, which often provide insufficient time for patient education.

In the experimental group, in addition to playing the video on a loop, the nurse issued brochures and provided oral explanations, thus deepening patients’ engagement with the educational content. Compared to the control group, the experimental group’s mode of health education clearly provided them with advantages in terms of learning methods, time, content, and other aspects. This is also an important reason for ultimately enhancing the patients’ knowledge of educational content.

### 4.2. Animated video-assisted postoperative health education can shorten the time spent on health education

This study’s results revealed that the time spent on health education in the experimental group was 3.51 ± 0.62 minutes, which was significantly less than the 6.76 ± 1.14 minutes in the control group. We believe this is related to the following factors.

First, the patients’ medical backgrounds were limited. All patients underwent infusion port implantation for chemotherapy, and most had no more than a high school level of education. This result is consistent with the findings of Tao et al.^[25]^ A low education level is directly linked to a patient’s inability to understand the content of oral or written expressions in a timely and adequate manner, and it may even lead to misunderstandings. Therefore, nurses had to repeatedly emphasize the content, which required a lot of time and energy to ensure that the patients understood the information accurately.

Second, animated videos have several advantages. With intuitive images, animations, and sounds, the video transformed the originally profound and complex medical knowledge into vivid graphic material that the patients could more easily comprehend.

In other words, patients in the experimental group already had a certain degree of understanding of the educational content by repeatedly watching the video before the nurses presented their health education. Therefore, even though the patients did not have a solid medical background, they were able to keep up with the pace and ideas without interrupting the nurses’ explanations or repeatedly asking questions to understand the information, making the education process smoother and less time-consuming.

## 5. Conclusions

The production of scientific, reasonable, and high-quality animated videos can compensate for the shortcomings of traditional health education. Animated videos can enhance the patients’ understanding of educational content and improve the efficiency of interventional nurses. This will have a multiplier effect on the development of postoperative health education after infusion port implantation. This study was conducted at 1 hospital’s invasive intervention center. We recommend that the reliability of our research results be further verified by expanding the sample size or combining multiple centers to examine the effects of video educational interventions.

## Author contributions

**Conceptualization:** Yan Zhang.

**Data curation:** Zhexia Jin, Zhongfeng Niu, Xi Hu.

**Formal analysis:** Zhexia Jin, Chunqiao Wu.

**Investigation:** Zhexia Jin, Chunqiao Wu, Funv Shen.

**Methodology:** Zhexia Jin, Funv Shen, Yayun Xiao.

**Software:** Zhongfeng Niu.

**Writing – original draft:** Zhexia Jin, Zhongfeng Niu.

**Writing – review & editing:** Yan Zhang.

## References

[1] Zhang Y Shi J Li JJ Zhang L Li Y. Systemic thrombolysis and anticoagulation therapy for catheter-related right atrial thrombosis caused by TIVAP: a case report and review of the literature. J Vasc Access. 2022;23:313–7. 33506722 10.1177/1129729821989159

[2] Rhu J Jun KW Song BJ Sung K Cho J. Cephalic vein approach for the implantable central venous access: a retrospective review of the single institution’s experiences; cohort study. Medicine (Baltim). 2019;98:e18007. 10.1097/MD.0000000000018007 PMC6867776 31725671

[3] Ye H Zeng J Qin W. A totally implantable venous access port associated with bloodstream infection caused by Mycobacterium fortuitum: a case report. Medicine (Baltim). 2018;97:e11493. 10.1097/MD.0000000000011493 PMC6086552 30024528

[4] Tao L Jin Y Jiang L. Economic analysis of port and PICC in long-term intravenous administration for malignant tumor patients in chinese oncology hospital setting. World J Integr Tradit Western Med. 2019;05:52–7.

[5] Moss JG Wu O Bodenham AR; CAVA trial group. Central venous access devices for the delivery of systemic anticancer therapy (CAVA): a randomised controlled trial. Lancet. 2021;398:403–15. 34297997 10.1016/S0140-6736(21)00766-2

[6] Zhejiang Implantable Venous Access Port Collaboration Group. Multidisciplinary consensus on clinical application of implantable venous access port(Zhejiang). J Pract Oncol. 2018;33:17–24.

[7] Zhang KC Chen L; Chinese Research Hospital Association Digestive Tumor Committee; Chinese Association of Upper Gastrointestinal Surgeons; Chinese Gastric Cancer Association and Gastrointestinal Surgical Group of Chinese Surgical Society Affiliated to the Chinese Medical Association. Chinese expert consensus and practice guideline of totally implantable access port for digestive tract carcinomas. World J Gastroenterol. 2020;26:3517–27. 32742123 10.3748/wjg.v26.i25.3517 PMC7366063

[8] Li CJ Wang BQ. Establishment of FTS-based health education path for chronic rhinosinusits patients in perioperative period. Nurs Res China. 2020;34:3827–33.

[9] Mahoney ST Tawfik-Sexton D Strassle PD Farrell TM Duke MC. Effects of education and health literacy on postoperative hospital visits in bariatric surgery. J Laparoendosc Adv Surg Tech A. 2018;28:1100–4. 29608433 10.1089/lap.2018.0093

[10] Downey D Graber K Lajoie D Newman L Weinstock P. Setting the stage: innovation in port access education for pediatric emergency nurses. J Emerg Nurs. 2023;49:631–9. 36872198 10.1016/j.jen.2023.01.002

[11] Piredda M Migliozzi A Biagioli V Carassiti M De Marinis MG. Written information improves patient knowledge about implanted ports. Clin J Oncol Nurs. 2016;20:E28–33. 26991720 10.1188/16.CJON.E28-E33

[12] Piredda M Biagioli V Giannarelli D. Improving cancer patients’ knowledge about totally implantable access port: a randomized controlled trial. Support Care Cancer. 2016;24:833–41. 26201750 10.1007/s00520-015-2851-1

[13] Poller B Hall S Bailey C. “VIOLET”: a fluorescence-based simulation exercise for training healthcare workers in the use of personal protective equipment. J Hosp Infect. 2018;99:229–35. 29421340 10.1016/j.jhin.2018.01.021 PMC7133760

[14] Breast Cancer Expert Committee of National Caner Quality Control Center. [Expert consensus on the whole-course management of implantable venous access port in the upper arm of cancer patients (2024 edition)]. Chin J Oncol. 2024;46:517–25. 10.3760/cma.j.cn112152-20231217-00364 38880733

[15] Chen L Zhang KC. Chinese expert consensus and practice guideline of totally implantable access port for digestive tract carcinoma (2019 edition). Chin J Pract Surg. 2020;40:152–7. 10.3748/wjg.v26.i25.3517 PMC7366063 32742123

[16] Aisah S Ismail S Margawati A. Animated educational video using health belief model on the knowledge of anemia prevention among female adolescents: an intervention study. Malays Fam Physician. 2022;17:97–104. 36606168 10.51866/oa.136 PMC9809444

[17] Supady A Nguyen K Aziz MAE Überreiter C Bärnighausen T Adam M. A short, animated storytelling video about sodium intake as a major cardiovascular risk factor and recommendations for a healthy diet: an online, randomized, controlled trial. Trials. 2023;24:390. 37296468 10.1186/s13063-023-07418-6 PMC10257297

[18] Platto JF Maarouf M Hendricks A Kurtzman DJ Shi VY. Animated video consultation for reducing pre-operative anxiety in dermatologic surgery. Dermatol Online J. 2019;25:1–7. 30982298

[19] Christie A Robertson I. A survey of nurse staffing levels in interventional radiology units throughout the UK. Clin Radiol. 2016;71:698–701. 27156208 10.1016/j.crad.2016.03.016

[20] Natcheva HN Silberzweig JE Chao CP. Survey of current status and physician opinion regarding ancillary staffing for the IR suite. J Vasc Interv Radiol. 2014;25:1777–84. 25161128 10.1016/j.jvir.2014.07.010

[21] Fan ZH Zhao YM Shi L. The influence of the application of health education tools on the acceptance degree and effect of patients during hospitalization. J Nurses Train. 2018;33:543–5.

[22] Glaser J Nouri S Fernandez A. Interventions to improve patient comprehension in informed consent for medical and surgical procedures: an updated systematic review. Med Decis Making. 2020;40:119–43. 31948345 10.1177/0272989X19896348 PMC7079202

[23] Greenlaw C Elhefnawy Y Jonas R Douglass LM. Using an animated video to promote an informed discussion on SUDEP with adolescents. Epilepsy Behav. 2021;122:108182. 34256339 10.1016/j.yebeh.2021.108182

[24] Schwarz U. [Health education through digital audio-visual media:strategies of the German Federal Centre for Health Education (BZgA)]. Bundesgesundheitsblatt Gesundheitsforschung Gesundheitsschutz. 2020;63:715–20. 32430510 10.1007/s00103-020-03145-4

[25] Tao QY Sun CX Zhang Y. Knowledge, belief, and practices of safety management of oral anticancer drugs among cancer patients. J Nurs Sci. 2020;35:27–30.

